# Panel of significant risk factors predicts early stage gastric cancer and indication of poor prognostic association with pathogens and microsatellite stability

**DOI:** 10.1186/s41021-021-00174-6

**Published:** 2021-02-10

**Authors:** Payel Chakraborty, Souvik Ghatak, Saia Chenkual, Lalawmpuii Pachuau, John Zohmingthanga, Zothankima Bawihtlung, Lalfakzuala Khenglawt, Jeremy L. Pautu, Arindam Maitra, Lalchhandama Chhakchhuak, Nachimuthu Senthil Kumar

**Affiliations:** 1 Department of Biotechnology, Mizoram University, Aizawl, Mizoram 796004 India; 2 Department of Surgery, Civil Hospital Aizawl, Aizawl, Mizoram 796001 India; 3 Department of Pathology, Civil Hospital Aizawl, Aizawl, Mizoram 796001 India; 4 Department of Radiation Oncology, Mizoram State Cancer Institute, Zemabawk, Aizawl, Mizoram 796017 India; 5 Department of Oncology, Mizoram State Cancer Institute, Zemabawk, Aizawl, Mizoram 796017 India; 6 National Institute of Biomedical Genomics, P.O. NSS, District Nadia, Kalyani, West Bengal 741251 India

**Keywords:** Gastric Cancer, Risk factors, *H. pylori*, *EBV*, MSI, Clinicopathological data

## Abstract

**Background:**

There are very few studies covering the epidemiological risk factors associated with *Epstein Barr Virus (EBV)* and Microsatellite stability for Gastric Cancer (GC) cases. Early diagnosis of GC through epidemiological risk factors is very necessary for the clinical assessment of GC. The aim of this study was to find out the major risk factors to predict GC in early stage and the impact of pathogen infection and MSI on survival rate of patients. GC samples were screened for *Helicobacter pylori, Epstein Barr Virus*, and Mismatch repair (MMR) gene status (microsatellite stable or instable). Chi-square and logistic regression analysis of Odd ratio and 95% confidence interval (OR, 95% CI) were performed to find out the association between epidemiological factors and the risk of gastric cancer. The pathogen and MMR gene status were analysed to predict their effect on overall survival and the risk score and hazard ratio was calculated for prognostic assessment.

**Results:**

Excess body weight, consumption of extra salt, smoked food, alcohol, and smoking were the major risk factors for GC development. This study achieved a high area under the curve (AUC 0.94) for the probable GC patients in early-stage using the five-panel epidemiological risk factors. *H. pylori* infected cases were significant with smoked food, while *EBV* was found to be associated with tuibur intake and smoked food. In overall survival analysis *EBV* infected and microsatellite stable (HR: 1.32 and 1.34 respectively) GC cases were showing poor prognosis.

**Conclusion:**

This study might provide new opportunities for personalized treatment options using this epidemiological factor risk score and clinicopathological factors assessment for early detection and prognosis in high-risk GC populations.

**Supplementary Information:**

The online version contains supplementary material available at 10.1186/s41021-021-00174-6.

## Introduction

Gastric Cancer (GC) is a heterogeneous disease and varies widely based on etiological factors and genetic architecture. Histologically, most of the GC are adenocarcinoma and can be further classified as diffuse (poorly differentiated) or intestinal (well-differentiated) types [1, 2], with unique epidemiological influence and genetic signatures. GC, being the fifth most commonly occurring cancer, is prevalent in the eastern and central parts of Asia and is the third most common cancer as per the mortality rate [3, 4]. Mizoram, Northeastern tribal state of India has the highest incidence rate of gastric cancer in India [5] and globally occupies the fifth position for GC [6].

Several studies reported that dietary, behavioral, and lifestyle habits significantly increase GC risk, and every population/ race has unique dietary and lifestyle habits. Mizo population also have unique traditional food and habit which might play role for developing GC. Mizo ethnic food, sa-um (fermented pork fat) is rich in fat content and has been shown to retain pathogens which can have an adverse effect on human health [7]. Sa-um preparation takes place on a cottage-industrial scale in households which does not have firmly established procedures and as a result the production process fluctuates on a seasonal basis. Another unique traditional habit of use of alkaline tobacco infused water (tuibur) containing polyaromatic hydrocarbons and carbonyl compounds [8, 9] may also have an effect on pathogen incidence as well as GC. Various studies suggest that there is an association between pathogens (*Helicobacter pylori* and *Epstein Barr Virus*) and microsatellite instablilty (MSI) for GC development [10–12]. The prevalence of pathogens and MSI associated GC cases varies depending on different populations [13, 14]. Few studies have highlighted about the risk factors of MSI in other cancers [15–19].

Till date, there is no in-depth study on the epidemiological risk factors associated with *Epstein Barr Virus (EBV)* and MSI for GC cases. Therefore, this study was carried out to find the unique risk factors which might be involved for developing pathogen and MSI associated GC. There is a lack of epidemiological markers to predict GC at an early stage and there is not much information available about the pathogen specific risk factors and their prognosis assessment on associated GC cases.

The aim of the present study is to: i) to find the predictive epidemiological factors which can aid to estimate the GC risk at an early stage, ii) to assess the significant risk factors which can elevate GC risk in presence of pathogens and MSI, and iii) also to assess the effect of pathogen infection and Mismatch repair (MMR) gene status on survival rate of patients. We hypothesize that the exposure to major risk factors can predict GC at an early stage and individuals with pathogens or MSI have increased risk of developing GC that might affect the survival rate of the patients.

## Materials and methods

### Study population

**Fig. 1:**
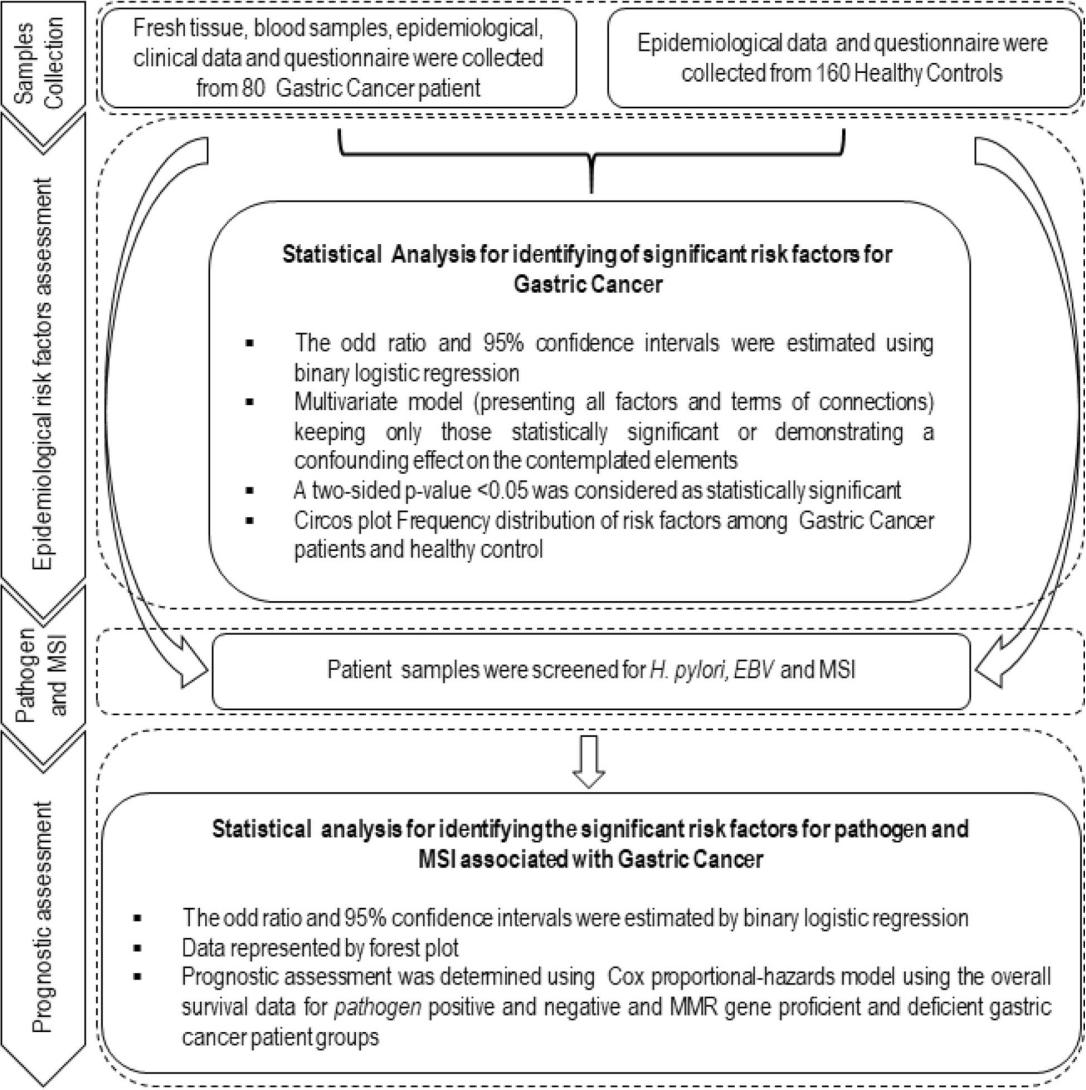
Study design for the epidemiological risk factors and prognostic assessments for H. pylori, EBV and MMR gene status among gastric cancer patient group

This is a case-control study consisting of GC patient samples collected from different hospitals (Civil Hospital Aizawl, Ebenezer Hospital, Aizawl Hospital, and Green Wood Hospital) in Mizoram, Northeast India from September 2016 to January 2019. The controls and cases were randomly selected at 2:1 ratio by age and sex, respectively. A total of 80 patients (53 male and 27 female) were selected after conforming histologically as a case of stomach adenocarcinoma by the pathologists. Their age ranged from 31 to 86 years. Patients who had any chronic diseases without GC, history or present record of gastritis and pre-treated for any other type of cancer were not eligible for this study. A total of 160 healthy controls (79 male and 81 female) were randomly selected from the same ethnic group from where the patients were selected and belong with almost similar age from 31 to 85 (57.96 ± 11.48) years. Patients who had any chronic diseases, gastritis, and cancer were not eligible as control. The work was approved by ethical committees of Civil Hospital, Aizawl (B.12018/1/13-CH(A)/IEC dtd. 18/04/2014), and Human Ethical Committee, Mizoram University (MZU/IHEC/2015/008 dtd. 14/12/15). The details study design was represented in Fig. 1.

### Data collection

All the participants of this study were interviewed using a well-designed and informative questionnaire with a duly informed consent form. A telephonic interview was also done for the follow-up study, with the patient group and respective clinicians. The questionnaire contains demographic information (age, sex and BMI), lifestyle habits like smoking (categorized as smokers and non-smokers), smokeless tobacco Chewed tobacco, Paan with betel nut, tuibur (tobacco infused water), a unique habit of Mizoram (categorized as consumers and non-consumers), alcohol (categorized as drinkers and non-drinkers), food habits like extra salt consumption, smoked food and saum, fermented pork fat (categorized as consumers and non-consumers). The clinical data like tumor size, anatomy, pathological TNM staging (American Joint Committee on Cancer, 8th edition: Tumor (T)- how deeply has the primary tumor spread into the stomach wall?; Node (N)- has the tumor spread to the lymph nodes and where and how many?; Metastasis (M)- has the cancer spread to other parts of the body?), tumor Grade, family history and overall survival status were also recorded using a structured questionnaire.

### DNA isolation from tissue and blood sample

Fresh gastric tumor tissue and peripheral blood samples (3 ml in EDTA Vial) for each patient and peripheral blood sample for healthy controls were collected. Genomic DNA was extracted from the cancerous tumor tissue and corresponding blood samples using commercially QIAamp® DNA Tissue Kit and QIAamp® Blood DNA mini kit. The extracted DNA was electrophoresised with 0.8% agarose gel and quantified using Picogreen dye in Qubit Fluorimeter (Invitrogen).

### Pathogen genotyping

The presence of *Helicobacter pylori* infection was determined in GC patients by PCR amplification of specific 16SrRNA region, *UraC* genes. The presence of *Epstein Barr Virus (EBV)* type1/ type 2 infection was carried out using a standard PCR assay by type-specific region (EBNA3C - Epstein–Barr virus nuclear antigen 3C) gene using specific primer sets [19]. The PCR reaction volume of 10 μl contained: 1x PCR buffer with, 1 unit of Taq DNA Polymerase, 0.2 mM dNTPs (All from the Thermo Scientific, USA), and 0.2 Pico mol primer (Active Oligo-ILS, Bangalore, India). The reaction mixture (10 μl) was PCR amplified for initial denaturation at 95 °C for 5 min, followed by 35 cycles at 95 °C for 1 min., n°C (depending on primer) for 40 s, 72 °C for 40 s/1 min followed by extension at 72 °C for 5 min. (Supplementary Table 1). *H. pylori* and *EBV* positive and negative control samples were used in all the PCR amplification for confirmation.

### PCR amplification of microsatellite loci

MSI was determined by comparison of the allelic profiles of the mononucleotide repeat markers BAT-25, BAT-26, and Dinucleotide Markers D2S123, D17S250, D16S752, D16S265, D16S398, D16S496, D18S58, and D16S3057 in tumor and corresponding blood and control blood 33-34 (Supplementary Table 1). The forward primers for the markers were labelled with fluorescent dye 6-FAM, VIC, NED, and PET. The PCR reaction volume of 10 μl contained: 1x PCR buffer, 1 unit of Taq DNA Polymerase, 0.2 mM dNTPs, and 0.15 Pico mol primers (Thermo Scientific). PCR was performed with a Master cycler (Eppendorf, nexus GX2). The following cycling regime was used as a “standard” PCR protocol: initial denaturation at 95 °C for 10 min, followed by 35 cycles at 94 °C for 1 min, 55 °C for 40 s and 72 °C for 40 s and the final extension step of 7 min at 72 °C (Supplementary Table 1).

### Fragment analysis

The amplified loci were analyzed using the automated ABI sequencer model 3500 Genetic Analyzer (Applied Biosystems, Singapore). In brief, 8.7 μl deionized formamide was combined with 0.3 μl GeneScan^Tm^-600 size standards (Applied Biosystems, V-2.0) and 1 μl PCR product in a Genetic Analyzer sample plate. After adding samples, the plate was sealed by septa, and mixing was done by mild vortexing. The denaturation step was done at 90 °C for 2 min, followed by keeping the plate on ice, and a mini-centrifugation for 1 min. The MSI of the investigated loci was defined as allele shift or (and) appearance of novel peaks. Samples were classified as MSI or MMR deficient if at least two or more than two markers were having instability and the instability was found only in BAT-26 Maker. If instability was not found in any of the markers, then the sample was classified as MSS [20] (Supplementary Figure 1).

### Statistical analysis

Distribution of demographic and lifestyle characteristics between the control and case groups were compared by chi-square test [21]. The odd ratio (OR) and 95% confidence intervals (CIs) were estimated for determining association in each group of factors among case-control subjects by binary logistic regression (Univariate and Multivariate analysis) [19]. All the demographic factors were grouped as follows: excess body weight [body mass index (BMI) ⩾ 25], Lifestyle habits such as: a) smoking, categorised as smokers (who used to smoke at least once a week for 3 months or more) and non-smokers (if the person never smoked before or left smoking for more than 5 years); b) chewing tobacco in smokeless form, categorised as consumer (who used to take atleast once a week for 6 months or more) and non-consumer (if the person never consume before or left more than 5 years before); c) tuibur or tobacco infused water, categorised as drinkers (if the person used to drink at least once in a week) and non-drinkers (if the person never drink); and d) alcohol, categorised as drinkers (if the person used to drink at least 1 day in a week) and non-drinkers (if the person never drink). It has detailed information on food habits such as: a) extra salt intake, categorised as consumers (if the person takes extra salt at least for once in their meal in a week) and as non-consumers (if the person never takes extra salt with their daily food for once); b) smoked food, categorised as consumers (if the person ate at least for 1 day in a week) and as non-consumers (if the person did not eat even for a single day in a week); and c) sa-um or fermented pork fat, categorised as consumers (if the person ate at least for once in a week) and as non-consumers (if the person did not eat even for once in a week).

The independent impact of hazard components was further explored in a multivariate model (presenting all factors and terms of connections) keeping only those statistically significant or demonstrating a confounding effect on the contemplated elements. The likelihood test was utilized to choose whether to hold each covariate in the model. BMI, Cigarette smoking, alcohol, smoked food (meat or vegetable consumption), high intake of salt were considered altogether in the estimated risk model as potential confounders to assess the relationship of hazard factors and susceptibility to gastric cancer. For all tests, a two-sided *p*-value < 0.05 was considered as statistically significant. Circos plot was generated using circos software for association demographic factors between GC patients and healthy control. Another association approach was done within the patients between the risk factors and clinical data among the subgroups of with or without *H. pylori*, *EBV* infection and MMR deficient (MSI)/MMR proficient (MSS) were estimated by calculating odds ratio (OR) and 95% confidence intervals (CIs) using binary logistic regression method and representing by forest plot using R software. Overall survival was determined using the Cox proportional-hazards regression model (using 3 years cut-off). The log-rank test, Kaplan-Meier survival analyses were used to assess the impact of the variables on survival. Variable used for survival analysis were *H. pylori* status, *EBV* status, MSI status, and anatomical site.

## Results

The baseline characteristics of the total GC patient cohort are presented in Supplementary Table 2. The age group interval of 40-69 years shows the highest number of GC patients (75%) in this cohort. About 32.5% of patients were having a first-degree family history of all types of cancer. Among the 80 GC patients, 50% of the cases were found in stage III and 8.75% were graded as well-differentiated, 46.25% were moderately differentiated and 32.5% were poorly differentiated cases. Most of the tumor was located in the distal part of the stomach and the prevalence of GC was high in male patients in this cohort (Supplementary Table 2).

**Fig. 2:**
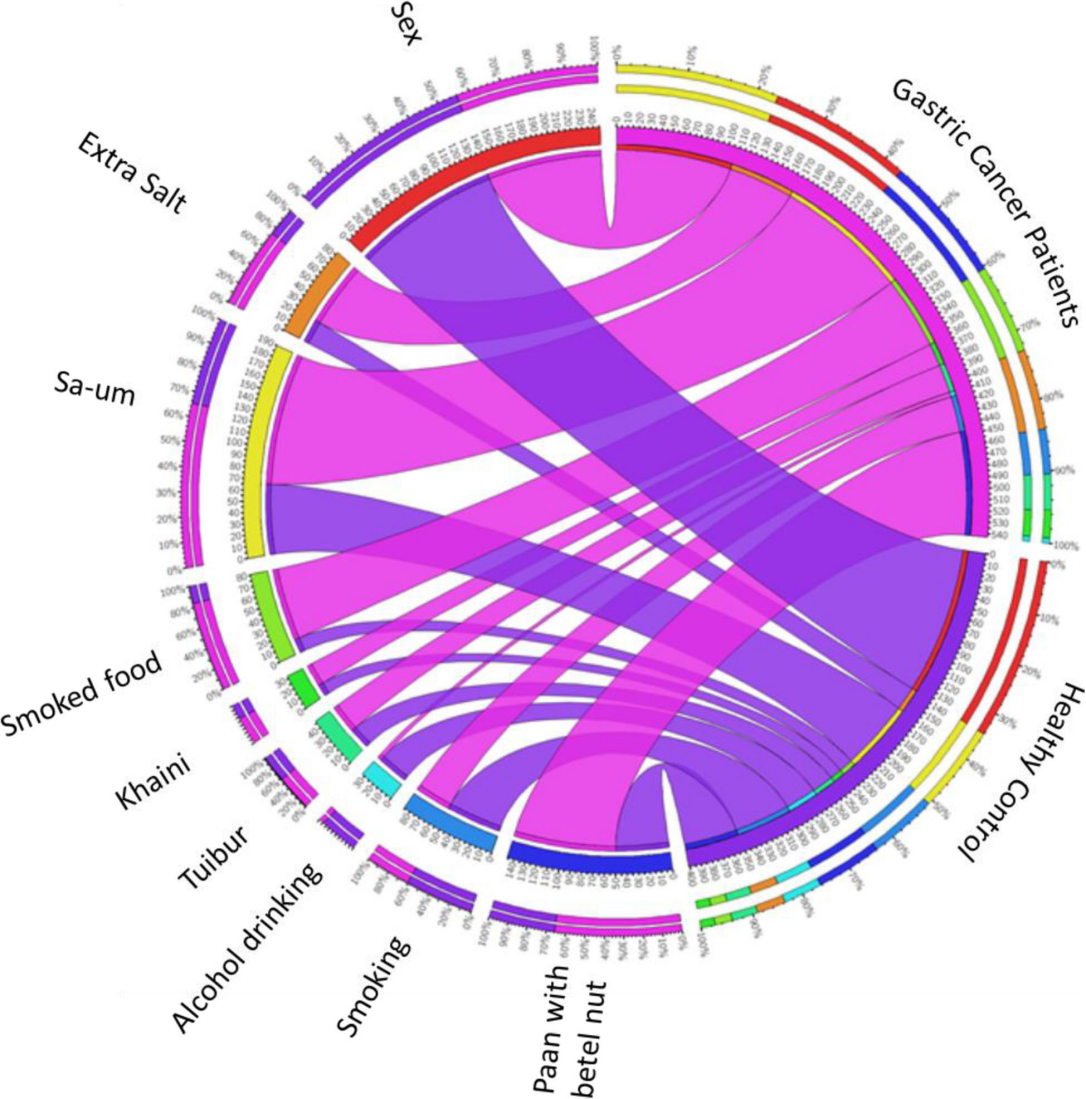
Frequency distributions of each demographic factors in the gastric cancer patients (pink ribbon) and healthy control (blue ribbon) groups in study cohort. The data were visualized via Circos software. The frequency of occurrence of different demographic factors association with gastric cancer and heathy control groups is depicted in the outer ring. The inner ring of circos plot depicts the subject number exposed with different demographic risk factors. Each factor has been assigned a specific color. The arc originates from gastric cancer and healthy control groups and terminates at different demographical factors to compare the association between the origin and terminating factors. The area of each colored ribbon depicts the frequency of the samples

Supplementary Table 3 presents the distribution of demographic and lifestyle habits among GC patient and controls. Extra salt consumption was a significant risk factor (*p* value = 0.0001) along with Smoked food consumption (*p* value = 0.01), Smoking (*p* value = 0.0001) and alcohol drinking (*p* value = 0.0001) which are also high risk factors for developing GC. The frequency and association of demographic factors and lifestyle habits between GC patients and healthy control (HC) were presented as Circos plot (Fig. 2).

The univariate binary logistic regression analysis was performed for sex, BMI, dietary and lifestyle habits.). Sex (*p-*value = 0.019) and BMI (*p-*value = 0.0001) were significant factors for the gastric cancer patients (Table 1). Among the dietary factors, extra salt consumption (*p-*value = 0.007) and smoked food consumption (*p-*value = 0.0001) were the major risk factors for the GC patients. Smokeless tobacco (tuibur) intake (*p-*value = 0.011), smoking (*p-*value = 0.0001) and alcohol consumption (*p-*value = 0.0001) became significant lifestyle risk factor for GC risk (Table 1).

**Table 1:** Univariate and multivariate analysis of the risk factors compared between Gastric Cancer patients (*n* = 73) and Healthy Controls (*n* = 153)

Factors	ODDS ratio (95% CI)	***p*** value
**Univariate analysis**		
Sex	0.50 (0.28 – 0.89)	0.019
Age	1.01 (0.99 – 1.04)	0.07
BMI	0.63 (0.56 – 0.72)	0.0001
Extra Salt	0.59 (0.41 – 0.86)	0.007
Sa-um	0.75 (0.50 – 1.13)	0.180
Smoked Food	0.49 (0.34 – 0.70)	0.0001
Tuibur	1.48 (1.09 – 2.00)	0.011
Alcohol drinking	3.11 (1.96 – 4.92)	0.0001
Smoking	7.50 (4.03 – 13.94)	0.0001
Paan with betel nut	0.99 (0.56 – 1.76)	0.984
**Multivariate analysis (logistic model)**		
Sex	0.58 (0.24 – 1.40)	0.230
BMI	0.69 (0.60 – 0.79)	0.0001
Extra Salt	0.68 (0.41 – 1.14)	0.042
Smoked Food	0.64 (0.40 – 1.04)	0.001
Tuibur	1.30 (0.80 – 2.12)	0.285
Alcohol drinking	1.83 (1.03 – 3.26)	0.001
Smoking	4.41 (1.86 – 10.43)	0.0007

**Fig. 3:**
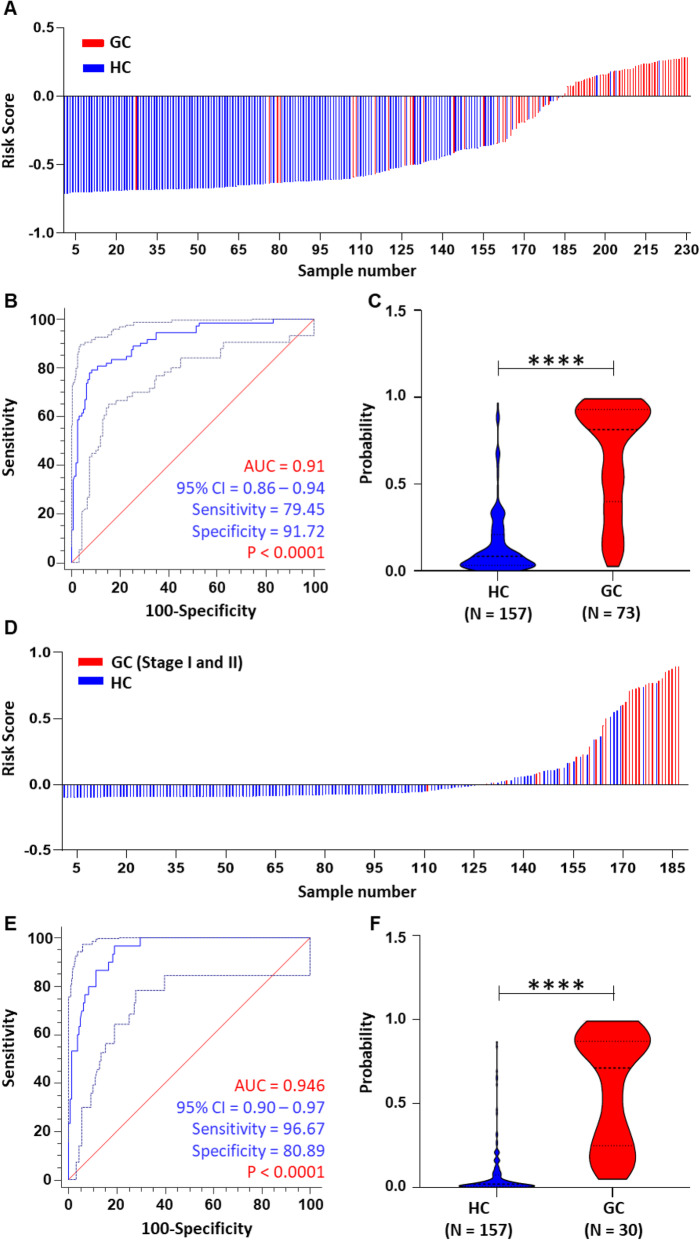
Estimation of accuracy value of the significant epidemiological factors based on the logistic model between gastric cancer and healthy control samples **a** Water fall plot and risk score estimation for stage-I, II, III and IV samples, **b** Receiver operating curve (ROC) and accuracy estimation of epidemiological factors panel (BMI, extra salt consumptions, smoked food consumptions, alcohol drinking and smoking) **c** Significant association of the estimated probability values of the epidemiological factors panel between gastric cancer (*n* = 73) and healthy controls (*n* = 157), **d** Water fall plot and risk score estimation for stage-I and II samples, **e** Receiver operating curve (ROC) and accuracy estimation of epidemiological factors panel. **f** Significant association of the estimated probability values of the epidemiological factors panel between stage-I and II gastric cancer (*n* = 30) and healthy controls (*n* = 157)

We further performed the multivariate analysis with these seven significant factors for finding out the major risk factors and confounding factors which were associated with GC development. Five factors were predicted as significantly associated with GC risk with high OR and 95% CI in multivariate analysis. BMI (*p-*value = 0.0001), Extra salt consumers (*p-*value = 0.042), smoked food consumers (*p-*value = 0.001), smokers (*p-*value = 0.0007) and alcohol drinkers (*p-*value = 0.001) were the high-risk groups associated with GC development (Table 1, Fig. 3a,). A risk score was estimated with the 5 factors using a logistic model and validated the risk score in the GC clinical cohort (Stage I, *N* = 20; Stage II, *N* = 14; Stage III, *N* = 40; Stage IV, *N* = 6) (Fig. 3a). The exposure to five-panel epidemiological factors might be successful in predicting the GC risk with different early symptoms (area under the curve – AUC = 0.91; *p-*value < 0.0001) (Fig. 3b). This 5-panel epidemiological factor achieved high-risk score with significant-high positive probability values for GC patients with high sensitivity (79.45%) and specificity (91.72%) (Fig. 3c).

For predicting GC at early-stage, a risk score was estimated with the 5 factors using a logistic model and was validated in the early stage (Stage I, *N* = 20 and II, *N* = 14) GC clinical cohort (Fig. 3d). The exposure of five-panel epidemiological factors might be successful in predicting the GC risk during the premalignant stage with different early symptoms with higher AUC value (0.946; *p-*value < 0.0001) (Fig. 3e). This 5-panel epidemiological factor achieved high-risk score with significant-high positive probability values for GC patients with high sensitivity (96.67%) and specificity (80.89%) (Fig. 3f). The estimated significant factors (BMI, extra salt consumption, smoked food, alcohol drinking, and smoking) were the major risk factors associated with GC development.

We have subdivided our GC patient cohort for pathogen infections and mismatch repair (MMR) gene deficiency with molecular identification of *H. pylori*, *EBV*, and MSI. Out of 80 patients, 71 (88.75%) cases were positive for the pathogens. Fifty cases (70.04%) were detected positive for *H. pylori, EBV* positive cases were 32 (45.07%) and a total of 11 (13.75%) gastric cancer patients were positive for both *H. pylori* and EBV. Moreover, 40% of cases were detected as MMR deficient (microsatellite instable-MSI) (Table 2, Supplementary Figure 1A) and 60% cases were detected as MMR proficient (microsatellite stable-MSS) (Table 2, Supplementary Figure 1B).

**Table 2:** Distribution of clinical factors among the various sub-groups in the gastric cancer patients’ cohort (*n* = 80)

Factors	***H. pylori*** (+) cases (***n*** = 50)	***H. pylori*** (−) cases (***n*** = 30)	***EBV*** (+) cases (***n*** = 32)	***EBV*** (−) cases (***n*** = 48)	MMR gene deficient (***n*** = 32)	MMR gene proficient (***n*** = 48)
**Anatomy**						
Proximal	8 (16%)	3 (10%)	4 (12.5%)	7 (14.58%)	3 (9.37%)	8 (16.66%)
Distal	35 (70%)	24 (80%)	21 (65.62%)	38 (79.16%)	28 (87.5%)	31 (64.58%)
Data Not available	7 (14%)	3 (10%)	7 (21.87%)	3 (6.25%)	1 (3.12%)	9 (18.75%)
**TNM Stage**						
I	11 (22%)	9 (30%)	8 (25%)	12(25%)	9(28.12%)	11(22.91%)
II	9 (18%)	5 (16.66%)	5 (15.62%)	9 (18.75%)	5 (15.62%)	9 (18.75%)
III	24 (48%)	16 (53.33%)	17 (53.12%)	23 (47.91%)	17 53.12%)	23 (47.91%)
IV	2 (4%)	0	1 (3.12%)	1 (2%)	0	2(4.16%)
Data Not available	4 (8%)	0	1 (3.12%)	3(6.25%)	1(3.12%)	3(6.25%)
**Grade**						
^a^WD	4 (8%)	3 (10%)	2 (6.25%)	5(10.41%)	2(6.25%)	5(10.41%)
^b^MD	23 (46%)	15 (50%)	12 (37.5%)	26 (54.16%)	12 (37.5%)	26 (54.16%)
^c^PD	20 (40%)	11 (36.66%)	16 (50%)	15 (31.25%)	17 (53.12%)	14 (29.16%)
Data Not available	3 (6%)	1 (3.3%)	2 (6.25%)	2(4.16%)	1(3.12%)	3(6.25%)
**Family history of Cancer**						
Yes	13 (26%)	14 (46.66%)	12 (37.5%)	15 (31.25%)	13 (40.62%)	14 (29.16%)
No	37 (74%)	16 (53.33%)	20 (62.5%)	33 (68.75%)	19 (59.37%)	34 (70.83%)

^a^*WD* Well Differentiated, ^b^*MD* Moderately Differentiated, ^c^*PD* Poorly Differentiated

We categorized our cases as *H. pylori* (+), *H. pylori* (−), *EBV* (+), *EBV* (−), MMR deficient, and MMR proficient and compared each group with clinical, demographic and lifestyle habit data to find out significant factor with each subgroup of GC patients. Table 2 presents the frequency distribution of clinical factors among the subgroups of GC patients. The tumor was located at high frequency in the distal portion of the stomach for the MMR deficient (87.5%) and *H. pylori*-positive (70%) patients group whereas less frequency was observed for *EBV* positive (65.62%) patient group. MMR deficient, *EBV* positive and *H. pylori*-positive cases were high for the poorly differentiated adenocarcinoma group whereas MMR proficient, *EBV* negative, and *H. pylori*-negative cases were high for the moderately differentiated adenocarcinoma patient group.

Smoked food consumption was the only significant risk factor associated with *H. pylori* positive GC patients and *EBV* infected patient group with respective *p* value (*p*-value = 0.006 and *p*-value = 0.002). Smokeless tobacco (tuibur) consumers (*p*-value = 0.06) were at low risk for developing *EBV* associated GC (Table 3). Tobacco chewing and Alcohol drinking were found as significant risk factor for MMR deficient patients group with high OR, 95% CI (*p*-value = 0.04) and (*p*-value = 0.03), respectively (Table 3).

**Table 3:** Distribution of demographic factors and lifestyle habits among the various sub-groups in the gastric cancer patients’ cohort (*n* = 80), ORs - ODDS Ratios

Factors	***H. pylori*** (+) cases (***n*** = 50)	***H. pylori*** (−) cases (***n*** = 30)	EBV (+) cases (***n*** = 32)	EBV (−) cases (***n*** = 48)	MMR gene deficient (***n*** = 32)	MMR gene proficient (***n*** = 48)
**Age (mean)**	59.5 ± 12.37	59.5 ± 9.76	59.5 ± 9.94	59.5 ± 12.36	56.5 ± 12.31	60 ± 10.60
**Sex**						
Male	34 (68%)	19 (63.33%)	20 (62.5%)	33 (68.75%)	12 (37.5%)	31 (64.58%)
Female	16 (32%)	11 (36.66%)	12 (37.5%)	15 (31.25%)	20 (62.5%)	17 (35.41%)
**Extra salt**						
Consumers	36 (72%)	20 (66.66%)	20 (62.5%)	36 (75%)	22 (68.74%)	34 (70.83%)
Non-consumers	14 (28%)	10 (33.33%)	12 (37.5%)	12 (25%)	10 (31.25%)	14 (29.16%)
ORs (95% CI), *p* value	1.32 (0.49 – 3.51); 0.57		0.55 (0.21 – 1.46); 0.23		0.90 (0.34 – 2-39); 0.84	
**Sa-um**						
Consumers	42 (84%)	24 (80%)	25 (78.12%)	41 (85.41%)	29 (90.62%)	37 (77.08%)
Non- consumers	8 (16%)	6 (20%)	7 (21.87%)	7 (14.58%)	3 (9.37%)	11 (22.91%)
ORs (95% CI), *p* value	1.31 (0.40 – 4.23); 0.64		0.60 (0.19 – 1.94); 0.40		2.87 (0.73 – 11.26); 0.12	
**Smoked food**						
Consumers	26 (52%)	25 (83.33%)	27 (84.37%)	24 (50%)	22 (68.74%)	29 (60.41%)
Non-consumers	24 (48%)	5 (16.66%)	5 (15.62%)	24 (50%)	10 (31.25%)	19 (39.58%)
ORs (95% CI), *p* value	**0.21 (0.07 – 0.65); 0.006**		**5.40 (1.78 – 16.37); 0.002**		1.44 (0.56 – 3.70); 0.44	
**Paan with betel nut**						
Consumers	30 (60%)	20 (66.66%)	21 (65.62%)	29 (60.41%)	23 (71.87%)	27 (56.25%)
Non-consumers	20 (40%)	10 (33.33%)	11 (34.37%)	19 (39.58%)	9 (28.12%)	21 (43.75%)
ORs (95% CI), *p* value	0.75 (0.29 – 1.93); 0.55		1.25 (0.49 – 3.17); 0.63		1.98 (0.76 – 5.18); 0.16	
**Chewed tobacco**						
Consumers	26 (52%)	15 (50%)	15 (46.87%)	26 (54.16%)	12 (37.5%)	29 (60.41%)
Non- consumers	24 (48%)	15 (50%)	17 (53.12%)	22 (52.08%)	20 (62.5%)	19 (39.58%)
ORs (95% CI), *p* value	1.08 (0.43 – 2.67); 0.86		0.74 (0.30 – 1.83); 0.52		**0.39 (0.15 – 0.98); 0.04**	
**Tuibur**						
Consumers	13 (26%)	8 (26.66%)	12 (37.5%)	9 (18.75%)	8 (25%)	13 (27.08%)
Non- consumers	37 (74%)	22 (73.33%)	20 (62.5%)	39 (81.25%)	24 (75%)	35 (72.91%)
ORs (95% CI), *p* value	0.96 (0.34 – 2.69); 0.94		2.60 (0.93 – 7.20); 0.06		0.89 (0.32 – 2.49); 0.83	
**Smoking**						
Smokers	35 (70%)	17 (56.66%)	19 (59.37%)	33 (68.75%)	21 (65.62%)	31 (64.58%)
Non-smokers	15 (30%)	13 (43.33%)	13 (40.62%)	15 (31.25%)	11 (34.37%)	17 (35.41%)
ORs (95% CI), *p* value	1.78 (0.69 – 4.57); 0.22		0.66 (0.26 – 1.68); 0.39		1.04 (0.40 – 2.67); 0.92	
**Alcohol drinking**						
Drinkers	17 (43%)	12 (40%)	10 (31.25%)	19 (39.58%)	16 (50%)	13 (27.08%)
Non-drinkers	33 (66%)	18 (60%)	22 (68.75%)	29 (60.41)	16 (50%)	35 (72.91%)
ORs (95% CI), *p* value	0.77 (0.30 – 1.97); 0.58		0.69 (0.26 – 1.78); 0.44		**2.69 (1.05 – 6.89); 0.03**	

**Fig. 4:**
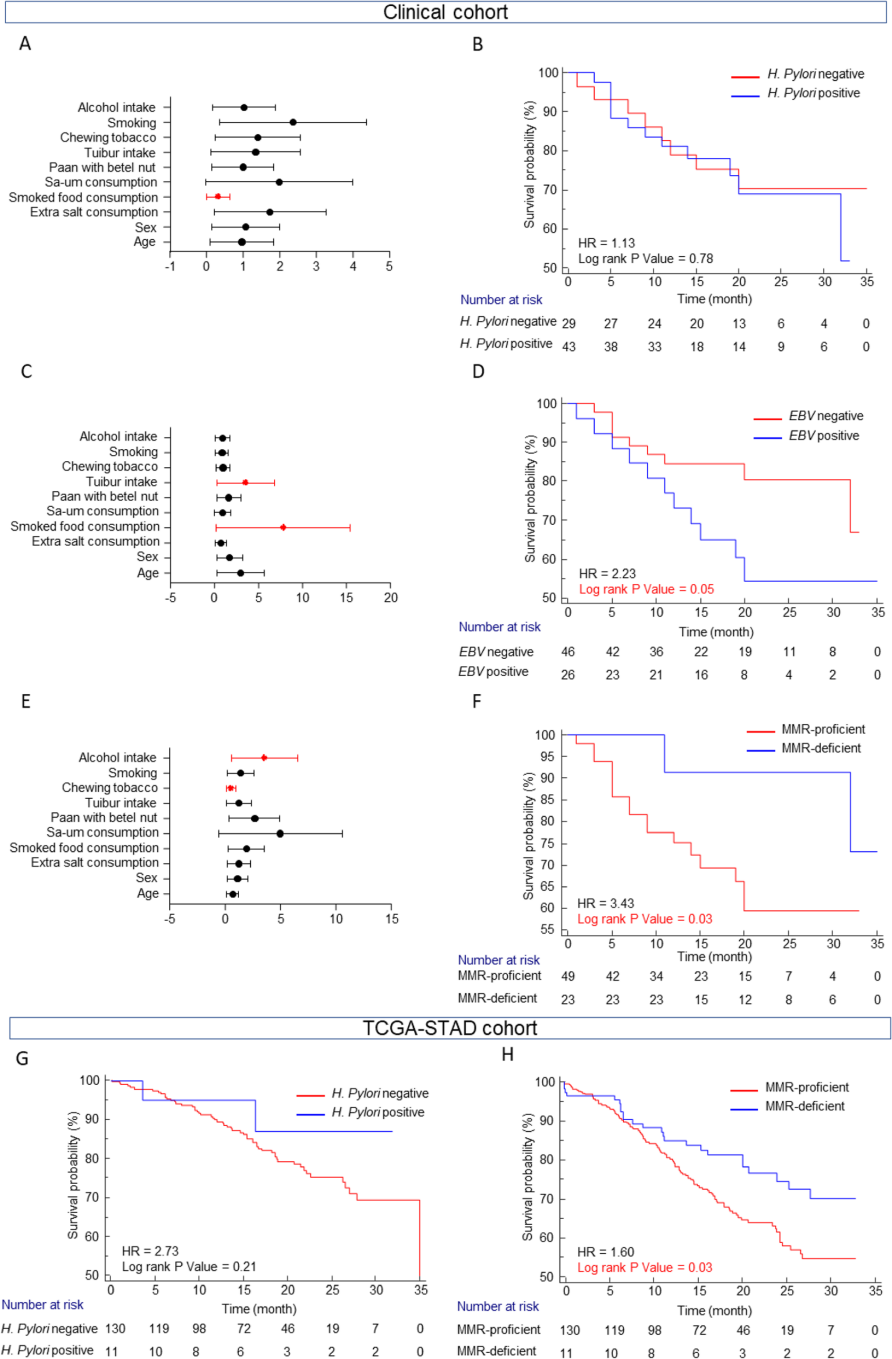
Association of overall survival probability and demographic factors with *H. pylori* infection, *EBV* infection and MMR gene status in gastric cancer patients. Odd ratios and 95% confidence interval of the demographic factors presented for the *H. pylori* (**a**), *EBV* (**c**) and MMR gene status (**e**). Association between overall survival and the *H. Pylori* (**g**) and MMR gene status (**h**) in TCGA-STAD cohort. *EBV* status could not be analyzed due to less sample size in TCGA-STAD dataset

For further verification, we performed binary logistic regression for determining the odd ratio and 95% CI. A significant association was found between *H. pylori*-infected GC patients with consumption of smoked food (*p*-value = 0.007) (Fig. 4a, Supplementary Table 4). Smoked food consumption (*p*-value = 0.003) and tuibur intake (*p*-value = 0.05) were significant factors for *EBV* infected GC patients and tuibur consumption (Fig. 4c, Supplementary Table 4). Significant association was observed with chewing tobacco (*p*-value = 0.04) and alcohol drinking (p-value = 0.03) for the MMR deficient (MSI) patient group (Fig. 4e, Supplementary Table 4). Factors such as smoked food and tuibur consumption are found to be the major risk for pathogen infection in GC patients and chewing tobacco, alcohol drinking as lifestyle factors became the risk factors for MMR deficient GC patients (Fig. 4e).

We further studied the overall survival (OS) rate of patients with the subgroup of with and without *H, pylori*, *EBV* infections, and MMR deficient and proficient patients to find out prognostic risk factors by unadjusted analysis after a follow-up of 36 months using the Kaplan Meier curve. A univariate Cox proportional hazards model demonstrated that there was no relation between *H. pylori* infections and GC patient’s prognosis with stage I, II, and III (HR: 1.13, 95% CI: 0.86 - 1.73; *p-*value = 0.13; Fig. 4b). *EBV* infections and MSI were an independent prognostic predictor for GC patients with stage I, II, and III (Fig. 4d and f). The *EBV* infected GC patients with stage I, II, and III was poor prognosis and high-risk patients (HR: 2.22, 95% CI: 0.92 - 2.97; *p-*value = 0.05). The comparison between MMR deficiency and proficiency exhibited significant prognostic predictor for stage I, II, and III GC patient groups (HR: 3.43; 95% CI: 0.95 - 4.08; *p-*value = 0.03). MSI/MMR deficient cases showed a good prognosis, whereas MSS/MMR proficient cases showed poor prognosis for GC patients (Fig. 4f). We have compared the *H. pylori*, *EBV,* and MMR gene status as independent prognostic factors for stage I, II, and III gastric cancer patients group in the TCGA-STAD cohort. Cox proportional-hazards regression model showed that there was no significant log-rank value *p-*value with *H. pylori* status (Fig. 4g) whereas MMR gene status an independent prognostic factor in TCGA-STAD cohort (HR: 1.60; 95% CI: 1.04 – 1.91; *p-*value = 0.03) (Fig. 4h).

## Discussion

To the best of our knowledge, this is the first retrospective study in Southeast Asia designed to assess the potential role of *H. pylori* / *EBV* infections, MMR gene status and epidemiological risk in the prognosis of GC patients. The results of the present study indicate that smoked food is the major risk factor that is significant in most of the subgroups of GC patients and the unique risk factor (tuibur) is found to be significantly associated with EBV infection. *EBV* infection is a strong risk factor for poor prognosis of GC in this Indian high-risk population.

Gender has significant impact for GC for this population. A large number of male gastric cancer patients (66.25%; OR = 0.50; 95% CIs = 0.28 – 0.89; *p*-value = 0.019) was found in our study. Excess body weight (BMI ⩾ 25) was associated with an risk of gastric cancer (OR = 0.63; 95% CIs = 0.56 – 0.72; *p*-value = 0.0001). Specifically, a multivariate stratified analysis showed that excess body weight was associated with an risk of gastric cancer [overweight and obese (BMI ⩾ 25), OR = 0.69, 95% CI = 0.60 – 0.79; *p*-value = 0.0001)]. Consumption of extra salt was found as dietary risk factor for GC. Extra salt can increase the mucin level of the surface mucus in the stomach which provides the possible condition for colonization of *H. pylori,* a significant risk factor of stomach cancer [22, 23]. It can significantly increase the carcinogenic A (CagA) gene expression in *H. pylori* which in turn alters the function of the epithelial cells and induces the hypergastrinemia in GC patients [24]. Extra salt intake could induce the inflammatory response of epithelial cells which may be responsible for cell proliferation and endogenous mutation [25] and moreover, it may increase susceptibility to the carcinogenic effects of N-nitroso compounds which can cause cell death [26]. Considering the present and past literature, we hypothesized that salt is a promoter of gastric adenocarcinoma, particularly in combination with *H. pylori* infection and that optimum quantity of salt consumption is significant for avoiding the gastric adenocarcinoma.

**Fig. 5:**
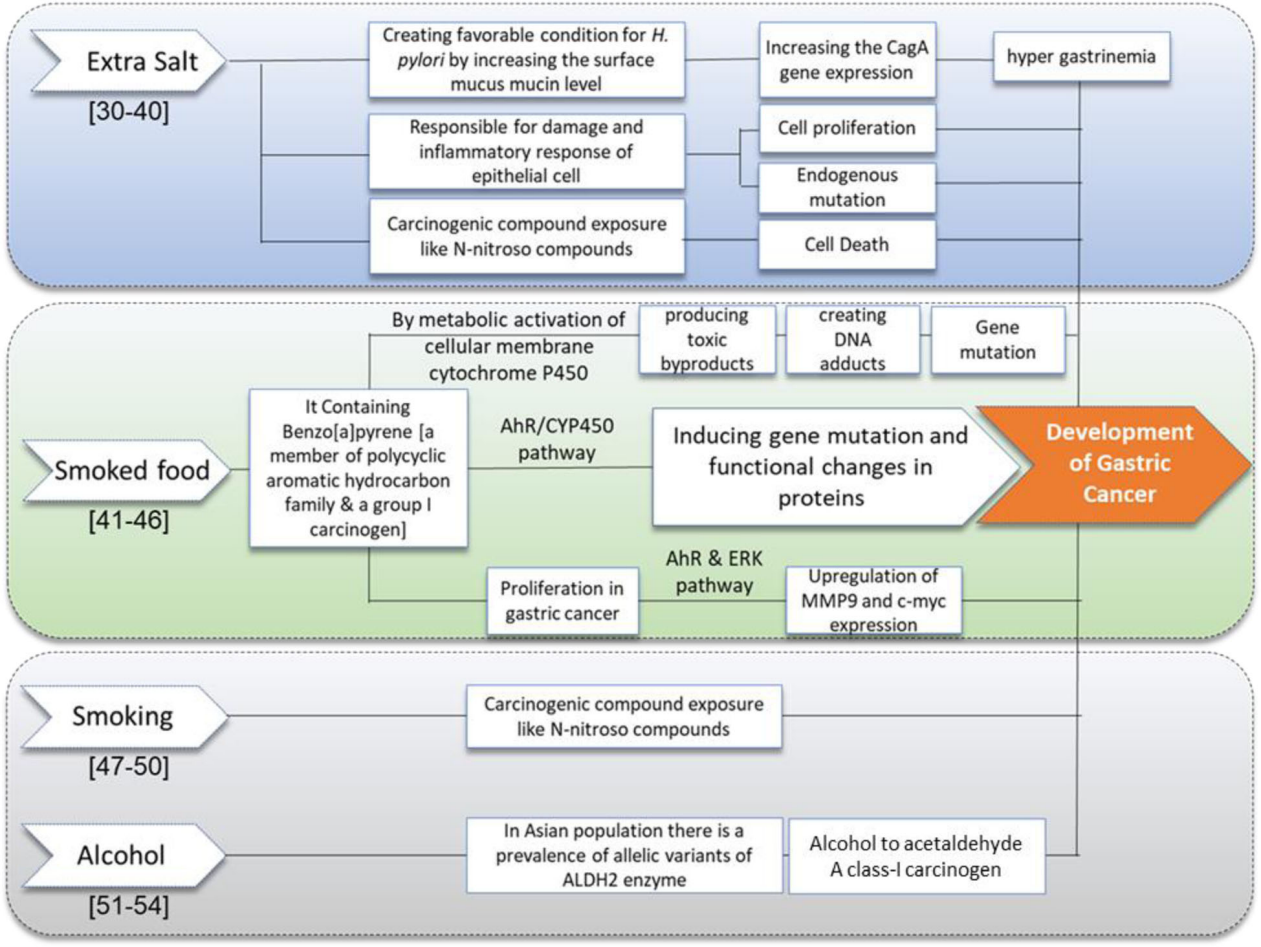
Flow chart depicting the Major risk factors in the present study and their mechanism of Gastric Carcinogenesis from Literature review. The numerical in parenthesis [] represents the bibliographic information

In this study, smoked food was found as another significant dietary risk factor with more than 60% of patients consuming the smoked food. Smoked food is generated by smoking or grilling method (a technique for cooking food on an oven or smoke generating system like burning of wood or charcoal) [27], and could produce both good antioxidants and antimicrobial properties, as well as carcinogenic chemicals like Polycyclic Aromatic Hydrocarbons (PAH) [28]. Benzo [a] pyrene (BaP), a member of PAH family, is a group I carcinogen which plays a role in the progression of GC and other cancers as well (Fig. 5). BaP accumulates in our body by metabolic activation of cellular membrane cytochrome P450 followed by producing toxic byproducts that will bind with DNA to create DNA adducts leading to gene mutation [29] and functional changes in proteins through AhR/CYP450 pathway [30]. BaP causes proliferation in GC cell lines and upregulation via*.* MMP9 and c-myc expression [31]. Studies have reported that smoked-dried or processed foods are strongly associated with GC development [19, 32] which is supporting our results.

In this study, smoking and alcohol consumption were also found as significant risk factors. Several studies have reported that smoking is an associated risk factor with GC [30, 33]. In this cohort, 65% of GC patients were smokers, whereas more than 78% were non-smokers in the healthy control group. Studies have reported that smoking has more impact on developing GC in men than in women [34], while another study has suggested that smoking is significant for both (men and women) to develop GC [35].

The effect of alcohol drinking on GC is always a matter of conflict. IARC has reported alcoholic beverages as a risk factor for humans in case of several cancers, but for GC no direct relations has been established so far [36], as most of the study showed uncertain results. ALDH2 is required to detoxify acetaldehyde which is a Class I carcinogen derived from alcohol by converting it to acetate. Mutations that inactivate ALDH2 are more prevalent in some Asian countries [37]. China has reported alcohol as an independent risk factor for GC in their population [38]. One Korean cohort study has reported that GC in the stomach cardia or upper third position had a significant association with smoking, and GC occurring in the distal part was associated with high alcohol consumption [39]. In our current study, more than 97% of healthy controls were non-drinkers, whereas more than 36% of patients were drinkers. One of the hypothetical mechanisms from the current and previous published study with all the significant risk factors for developing GC has been represented in Fig. 5.

We estimated the chances of GC development using the estimated risk score of the 5 different epidemiology factors (BMI, extra salt, smoked food, alcohol consumption, and smoking habit, Fig. 3a). The consumption of all the five factors is independently associated with GC risk in univariate analysis, whereas tuibur consumption did not achieve significance *p*-value in the multivariate model for GC risk. We found a significant difference in risk score probability between gastric cancer patients and healthy control (*p*-value < 0.0001, Fig. 3b). This study achieved a high area under the curve (AUC = 0.946) value for detecting the probable GC patients from the population using the 5-panel epidemiology factors at an early stage (Fig. 3c).

In the current study, a significant association was observed between smoked food consumption and *H. pylori* infection associated GC. Several studies reported the strong association between *H. pylori* and extra salt [22, 23]. Extra Salt-curing or brining adds flavor, allows the nitrites to penetrate the flesh and most important, extracts moisture from the food, allowing the smoke to penetrate more easily. Most cold-smoked meats are generally salt-cured or brined first. In this population, smoked foods are also rich in salt which can make a favorable condition for *H. pylori* infection and lead to developing the GC. Further studies with prospectively collected GC samples are necessary to support our data. In the present study, the consumption of smoked food was a significant risk factor for GC with *EBV* infection. Consumption of smoked food, smoking cigarettes are significant contributing factors, for developing carcinogenesis of GC, which might be amplified by the presence of *EBV*. It has been reported that smoking has a strong association with the risk of *EBV*-positive Hodgkin’s lymphoma [40] and that tobacco a risk factors for GC, may contain *EBV*-activating substances [41]. In the current study, tuibur consumption (tobacco infused water) was also a significant risk factor for *EBV* infected GC patients. Tuibur, a unique risk factor is tobacco infused water, so we can categorize it as smokeless tobacco and it contains polyaromatic hydrocarbons and carbonyl compounds. Studies have reported that smokeless tobacco affects B-lymphocytes [42], where latent *EBV* virus infection takes place [43] and infected lymphocytes at a later stage are responsible for tumorigenesis. Studies reported a positive association between smokeless tobacco and *EBV* type I and type II infections [44]. Another important aspect is *EBV* spreads by body fluids, especially saliva. In rural villages, the tuibur consumers share the same tuibur bottle for drinking and it can pass on from an *EBV* infected person to others through saliva. As smoked food preparation is done by exposing smoke and whole tobacco plant is used for tuibur preparation, so it can also help to increase the risk of *EBV* infected GC which needs to be revealed by further study.

This study has reported two lifestyle factors, chewing tobacco and alcohol drinking as a significant risk factor associated with MMR deficient GC patients. Studies have reported that tobacco and alcohol drinking are strongly associated with MSI-H colorectal cancer cases [15–19]. In our study, we found alcohol drinking and traditional tobacco (chewing tobacco) as a significant risk with MSI associated GC.

This study has claimed that *EBV* infected GC patients are showing poor prognosis and multivariate analysis has confirmed the prognostic value of *EBV* infection, even after adjustments for other clinical factors. The prognostic assessment for *EBV* associated GC is very much controversial as previous studies reported that median survival times for *EBV* associated GC (8.5 years) is higher, compared to *EBV*-negative tumor (5.3 years) [45] and 5 year OS in *EBV* associated GC (71.4%) is higher, compared to negative group (56.1%) [46]. The prognostic assessment for *EBV* associated GC is regionally and ethically restricted with their food and lifestyle habits. Moreover, there is a prevalence of *EBV* infected cases (45.07%) in this cohort compared to worldwide status (10%) [47], while *H. pylori* infection does not exhibit any significant change in survival rate associated with GC. In a study performed in china, a trend towards a higher survival rate in patients with high-copies *H. pylori* infection was observed compared to patients with low-copy infection [48]. MMR deficient GC patients exhibited good prognosis, while MMR proficient GC cases were considered as a high-risk group and more aggressive. The result is consistent with several studies reporting that MSI shows a better prognosis than MSS in gastric cancer [49–52]. Our prognostic assessments were comparable with TCGA data for the *H. pylori* and MSI patient groups (Fig. 4g and h). Our study also supports the fact that *H. pylori* infection is not a prognostic factor for GC patients for this population.

The prospective of this study is the panel of epidemiological risk factors which can predict GC at early stage, which is very necessary for making clinical decision on patient treatment. The prognostic assessment of this study will help clinicians to opt for the right therapy. Other strength of this study is the consistency in results obtained for the positive association between excess body weight (BMI), extra salt intake, smoked food and alcohol consumption, smoking and gastric cancer across high-quality studies with different patient populations. A limitation of this study is the smaller sample size, and further studies with large cohorts would be beneficial to support our data.

## Conclusion

This study reported significant etiological factors associated with GC development through multidisciplinary approaches. The study has augmented the literature on unique lifestyle risk factors associated with *EBV* infected patients and has identified a panel of five epidemiological risk factors to predict GC in early stages, which is necessary for better diagnosis and treatment of patients. This study gave an assessment on the survival of GC patients associated with pathogen and MMR gene status.

In conclusion, most of the cases are reported at an advanced stage which decreases the scope of treatment and resulting in poor survival rate. The risk score for 5-panel epidemiological factors, from the present study, could be used for predicting gastric cancer risk in the pre-malignant stage with an early symptom. Smoked food emerged as a major exposure for GC development and we can conclude that *EBV* infection is also the strong risk factor for gastric cancer mortality. In clinical practices, patients with curatively resected gastric cancer who are positive for *EBV* may need more careful follow-up and more aggressive antitumor treatment to prolong life expectancy. Further research is required to elucidate the exact mechanisms of inflammation and tumor suppression with or without pathogen infection, which might provide new opportunities for personalized treatment options using this risk score and clinicopathological factors.

## Supplementary Information

**Additional file 1: Supplementary Table 1.** Primer sequences used for screening of pathogens and MSI in Gastric cancer samples. **Supplementary Table 2.** Characteristics of Gastric Cancer patients in this study. **Supplementary Table 3.** Distribution of demographic and lifestyle habit factors among GC patient and controls, Sa-um- fermented pork fat. **Supplementary Table 4.** Univariate analysis of association of demographic factors with pathogens and MMR genes status in Gastric Cancer patients’ cohort. Both presence and absence of pathogens were taken into analysis. In case of MMR gene status Both MMR deficient and MMR proficient were taken into analysis.

**Additional file 2: Supplementary Fig. 1.** (A) Representing MSI case and (B) Representing MSS case. Here Comparison was done by Tumor and blood sample of studied patient.

**Additional file 3.**

## Data Availability

The datasets used or analyzed during the current study are available from the corresponding author on reasonable request.

## Abbreviations

GC: Gastric cancer
*EBV*: *Epstein bar virus*
*H.pylori*: *Helicobacter pylori*
MSI: Microsatellite Instability
MSS: Microsatellite stability
MMR: Mismatch repair
pTNM: Pathological. Tumour. Node. Metastasis
PAH: Polycyclic Aromatic Hydrocarbons
BaP: Benzo amino pyrene
TCGA-STAD: The Cancer Genome Atlas-Stomach Adenocarcinoma
ACRG: Asian Cancer Research Group
IARC: International Agency for Research on Cancer
AhR pathway: Aryl hydrocarbon receptor pathway
ERK pathway: Extracellular-signal-regulated kinase pathway
EMT: Epithelial-mesenchymal transition
HR: Hazard Ratio
CI: Confidence Interval
