# Supplementary material for: Panel of significant risk factors predicts early stage gastric cancer and indication of poor prognostic association with pathogens and microsatellite stability
Source: Genes Environ. 2021 Feb 10;43:3. doi: 10.1186/s41021-021-00174-6 (PMC7877109; doi:10.1186/s41021-021-00174-6)
Supplement: Supplementary file 1 — Additional file 1: Supplementary Table 1. Primer sequences used for screening of pathogens and MSI in Gastric cancer samples. Supplementary Table 2. Characteristics of Gastric Cancer patients in this study. Supplementary Table 3. Distribution of demographic and lifestyle habit factors among GC patient and controls, Sa-um- fermented pork fat. Supplementary Table 4. Univariate analysis of association of demographic factors with pathogens and MMR genes status in Gastric Cancer patients’ cohort. Both presence and absence of pathogens were taken into analysis. In case of MMR gene status Both MMR deficient and MMR proficient were taken into analysis. [file 41021_2021_174_MOESM1_ESM.docx]

| Primer sequences for *H. pylori* and *EBV* genotyping | | | | | | |
| --- | --- | --- | --- | --- | --- | --- |
| **Gene** | **Primer (5’to 3’)** | **Product Size (bp)** | | | **Annealing Temperature** | **Annealing Time** |
| 16SrRNA | F- CTGGAGAGACTAAGCCCTCC  R- ATTACTGACGCTGATTGTGC | 109 | | | 60°C | 40 sec |
| UraC | F: 5’ – AAGCTTTTAGGGGTGTTAGGGGTTT -3’  R: 5’ – AAGCTTACTTTCTAACACTAACGC -3’ | 294 | | | 54°C |  |
| EBNA 3C | F- AGAAGGGGAGCGTGTGTTGT  R- GGCTCGTTTTTGACGTCGGC | Type I- 153  Type II- 246 | | | 59°C |  |
| Microsatellite Markers used in the study | | | | | | |
| **Microsatellite Marker Name** | **Primer Sequences** | **Dye**  **5’ labelled in Forward primer** | **Marker Size (bp)** | **Repeat** | **Annealing**  **Temperature** | **Gene Name & Chromosome numbers** |
| BAT25 | F: 5’ -TCG CCT CCA AGA ATG TAA GT - 3’  R: 5’ - TCT GCA TTT TAA CTA TGG CTC - 3’ | PET | 110 - 133 | (T)25 | 56°C | KIT proto-oncogene receptor tyrosine kinase (KIT) , chromosome 4 |
| BAT26 | F: 5’ - TGA CTA CTT TTG ACT TCA GCC -3’  R: 5’ - AAC CAT TCA ACA TTT TTA ACC C -3’ | NED | 95 - 120 | (A)26 | 56°C | mutS homolog 2 (MSH2), chromosome 2 |
| D2S123 | F: 5’- AAA CAG GAT GCC TGC CTT TA - 3’  R: 5’ - GGA CTT TCC ACC TAT GGG AC -3’ | NED | 194 - 230 | (CA)29 | 59°C | DNA segment containing (CA) repeat, chromosome 2 |
| D17S250 | F: 5’ - GGA AGA ATC AAA TAG ACA AT - 3’  R: 5’ - GCT GGC CAT ATA TAT ATT TAA ACC - 3’ | VIC | 140 - 170 | (CA)19 | 52°C | DNA segment containing (CA) repeat, chromosome 17 |
| D16S752 | F: 5'-AATTGACGGTATATCTATCTGTCTG-3'  R: 5'-GATTGGAGGAG GGTGATTCT-3' | 6-FAM | 92-126 | (CTAT)11 | 57°C | CDH1, chromosome 16 |
| D16S265 | F: 5'-CCAGACATGGCAGTCTCTA-3'  R: 5'-AGTCCTCTGTGCAC TTTGT-3' | VIC | 95 - 115 | (CA)21 | 58°C | CDH1, chromosome 16 |
| D16S398 | F: 5'-CTTGCTCTTTCTAAACTCCA-3'  R: 5'-GAAACCAAGTGGGT TAGGTC-3' | PET | 175 - 195 | (CA)23 | 55.5°C | CDH1, chromosome 16 |
| D16S496 | F: 5’- GAAAGGCTACTTCATAGATGGCAAT-3’  R: 5’- ATAAGCCACTGCGCCCAT-3’ | VIC | 200 - 230 | (T)13 and (CA)21 | 61°C | CDH1, chromosome 16 |
| D18S58 | F: 5’-GCTCCCGGCTGGTTTT-3’  R: 5’- GCAGGAAATCGCAGGAACTT -3’ | 6-FAM | 140 - 155 | (CA)18 | 60°C | DNA segment containing (CA) repeat, chromosome 18 |
| D16S3057 | F: 5’-CCTGTGTGTATAACTATGTCAAAAT-3’  R: 5’-GCCCTTGAAACTAGGCAATA-3’ | 6-FAM | 190 - 207 | (CG)19 | 57°C | DNA segment containing (CG) repeat, chromosome 17 |

**Supplementary Table 1**: Primer sequences used for screening of pathogens and MSI in Gastric cancer samples

| **Factors** | **N (Total =80)** | **%** |
| --- | --- | --- |
| Median Age ± SD | 59.5 ± 11.40 |  |
| **Age (years)** |  |  |
| <40 | 1 | 1.25 |
| 40-69 | 60 | 75 |
| >69 | 19 | 23.75 |
| **Sex** |  |  |
| Male | 53 | 66.25 |
| Female | 27 | 33.75 |
| **Family History of Cancers** |  |  |
| Yes | 28 | 35 |
| 1^st^ degree relative | 26 | 32.5 |
| 2^nd^ degree relative | 2 | 2.5 |
| No | 52 | 65 |
| **Anatomy** |  |  |
| Distal | 59 | 73.75 |
| Proximal | 11 | 13.75 |
| Data Not available | 10 | 12.5 |
| **Stage** |  |  |
| I | 20 | 25 |
| II | 14 | 17.5 |
| III | 40 | 50 |
| IV | 2 | 2.5 |
| Data Not Available | 4 | 5 |
| **Differentiation** |  |  |
| Well Differentiated | 7 | 8.75 |
| Moderately Differentiated | 37 | 46.25 |
| Poorly Differentiated | 26 | 32.5 |
| Data Not available | 4 | 5 |

**Supplementary Table 2:** Characteristics of Gastric Cancer patients in this study

| **Factors** | **^a^HC (n = 160)** | **^b^GC (n = 80)** | **^c^ORs (95% CI)^d^** | ***p* value** | |
| --- | --- | --- | --- | --- | --- |
| **Age (Years ± SD)** | 57 ± 11.48 | 59.5 ± 11.40 | - | - |  |
| **Gender** | | | | |  |
| Male | 79 (49.37%) | 53 (66.25%) | - | - |  |
| Female | 81 (50.62%) | 27 (33.75%) |  |  |  |
| **Extra salt** | | | | |  |
| Consumers | 150 (93.75%) | 56 (70%) | 0.15 (0.07 – 0.34) | <0.0001 |  |
| Non-consumers | 10 (6.25%) | 24 (30%) |  |  |  |
| **Sa-um** | | | | |  |
| Consumers | 132 (82.5%) | 66 (82.5%) | 1.00 (0.49 – 2.02) | 1.00 |  |
| Non- consumers | 28 (17.5%) | 14 (17.5%) |  |  |  |
| **Smoked food** | | | | |  |
| Consumers | 126 (70%) | 51 (63.75%) | 0.47 (0.26 – 0.85) | 0.01 |  |
| Non-consumers | 34 (30%) | 29 (36.25%) |  |  |  |
| **Paan with betel nut** | | | | |  |
| Consumers | 97 (60.62%) | 50 (62.5%) | 1.08 (0.62 – 1.88) | 0.77 |  |
| Non-consumers | 63 (39.37%) | 30 (37.5%) |  |  |  |
| **Chewed tobacco** | | | | |  |
| Consumers | 63 (39.37%) | 41 (51.25%) | 1.61 (0.94 – 2.78) | 0.08 |  |
| Non- consumers | 97 (60.62%) | 39 (48.75%) |  |  |  |
| **Tuibur** | | | | |  |
| Consumers | 27 (16.87%) | 21 (26.25%) | 1.45 (0.91 – 3.35) | 0.08 |  |
| Non- consumers | 133 (83.12%) | 59 (73.75%) |  |  |  |
| **Smoking** | | | | |  |
| Smokers | 34 (21.25%) | 52 (65%) | 6.88 (3.79 – 2.48) | <0.0001 |  |
| Non-smokers | 126 (78.75%) | 28 (35%) |  |  |  |
| **Alcohol drinking** | | | | |  |
| Drinkers | 4 (2.5%) | 29 (36.25%) | 22.17 (7.44- 66.10) | <0.0001 |  |
| Non-drinkers | 156 (97.5%) | 51 (63.75%) |  |  |  |

**Supplementary Table 3:** Distribution of demographic and lifestyle habit factors among GC patient and controls, Sa-um- fermented pork fat.

| **Factors** | **OD (95% CI)** | ***p* value** |  |
| --- | --- | --- | --- |
| ***H. pylori*** | | | |
| Age | 0.70 (0.26 – 1.91) | 0.49 |  |
| Sex | 0.81 (0.31 – 2.10) | 0.66 |  |
| Extra salt consumption | 1.28 (0.48 – 3.42) | 0.61 |  |
| Smoked food consumption | **0.21 (0.07 – 0.65)** | **0.007** |  |
| Sa-um consumption | 1.31 (0.40 – 4.23) | 0.64 |  |
| Paan with betel nut consumption | 0.75 (0.29 – 1.93) | 0.55 |  |
| Tuibur intake | 0.96 (0.34 – 2.69) | 0.94 |  |
| Chewing tobacco | 1.08 (0.43 – 2.67) | 0.86 |  |
| Smoking | 1.78 (0.69 – 4.57) | 0.22 |  |
| Alcohol intake | 0.77 (0.30 – 1.97) | 0.58 |  |
| ***EBV*** | | | |
| Age | 2.14 (0.77 – 5.95) | 0.14 |  |
| Sex | 1.32 (0.51 – 3.38) | 0.56 |  |
| Extra salt consumption | 0.55 (0.21 – 1.46) | 0.23 |  |
| Smoked food consumption | **5.40 (1.78 – 16.37)** | **0.003** |  |
| Sa-um consumption | 0.61 (0.91 – 1.94) | 0.40 |  |
| Paan with betel nut consumption | 1.25 (0.49 – 3.17) | 0.63 |  |
| Tuibur intake | **2.60 (0.90 – 7.20)** | **0.05** |  |
| Chewing tobacco | 0.74 (0.30 – 1.83) | 0.52 |  |
| Smoking | 0.66 (0.26 – 1.68) | 0.39 |  |
| Alcohol drinking | 0.69 (0.27 – 1.78) | 0.44 |  |
| **MMR genes** | | | |
| Age | 0.48 (0.18 – 1.27) | 0.14 |  |
| Sex | 0.82 (0.32 – 2.15) | 0.70 |  |
| Extra salt consumption | 0.90 (0.34 – 2.39) | 0.84 |  |
| Smoked food consumption | 1.44 (0.56 – 3.70) | 0.44 |  |
| Sa-um consumption | 2.87 (0.73 – 11.26) | 0.13 |  |
| Paan with betel nut consumption | 1.98 (0.76 – 5.18) | 0.16 |  |
| Tuibur intake | 0.89 (0.32 – 2.49) | 0.83 |  |
| Chewing tobacco | **0.39 (0.15 – 0.98)** | **0.04** |  |
| Smoking | 1.04 (0.40 – 2.67) | 0.92 |  |
| Alcohol drinking | **2.69 (1.05 – 6.89)** | **0.03** |  |

**Supplementary Table 4:** Univariate analysis of association of demographic factors with pathogens and MMR genes status in Gastric Cancer patients’ cohort. Both presence and absence of pathogens were taken into analysis. In case of MMR gene status Both MMR deficient and MMR proficient were taken into analysis.
