# Supplementary material for: Panel of significant risk factors predicts early stage gastric cancer and indication of poor prognostic association with pathogens and microsatellite stability
Source: Genes Environ. 2021 Feb 10;43:3. doi: 10.1186/s41021-021-00174-6 (PMC7877109; doi:10.1186/s41021-021-00174-6)
Supplement: Supplementary file 2 — Additional file 2: Supplementary Fig. 1. (A) Representing MSI case and (B) Representing MSS case. Here Comparison was done by Tumor and blood sample of studied patient. [file 41021_2021_174_MOESM2_ESM.docx]

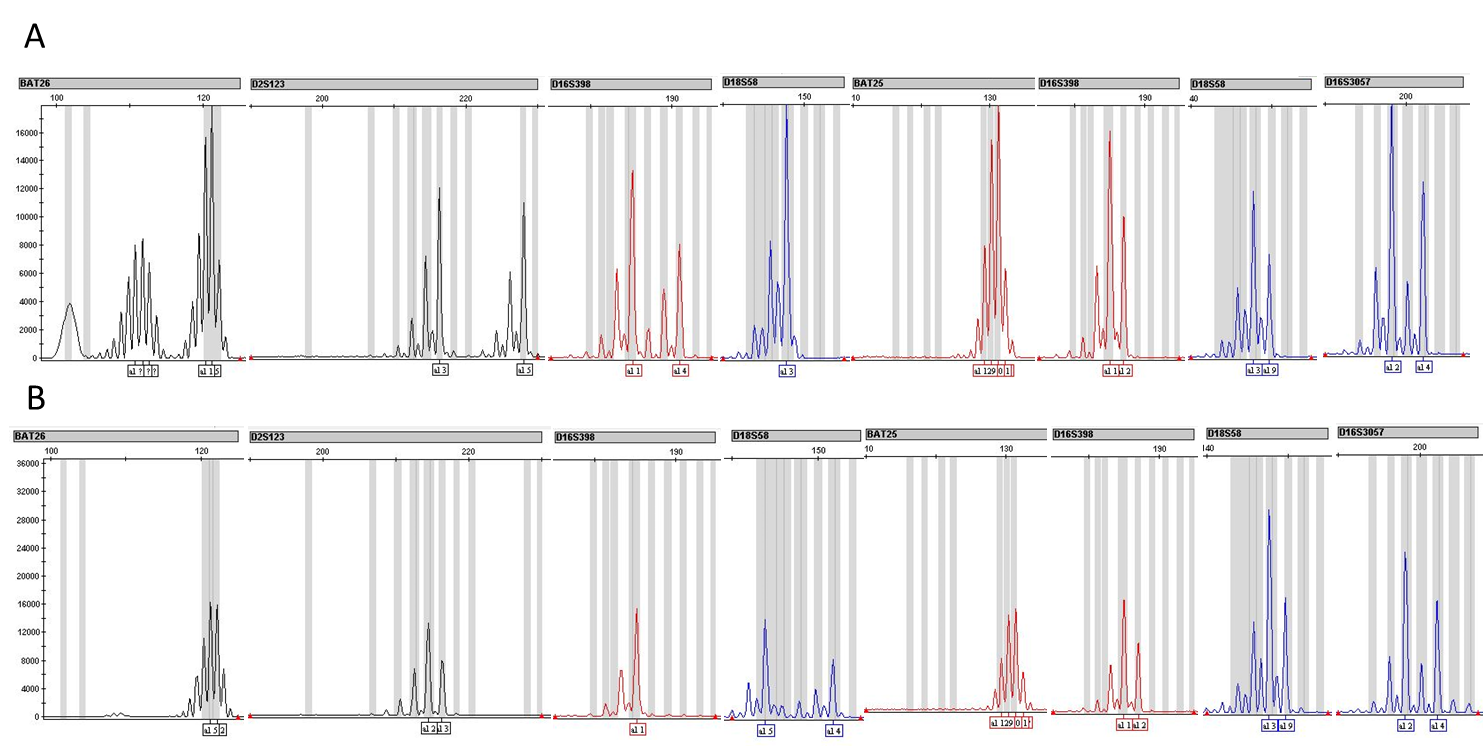


Supplementary Fig 1: (A) Representing MSI case and (B) Representing MSS case. Here Comparison was done by Tumor and blood sample of studied patient.
