# Supplementary material for: Panel of significant risk factors predicts early stage gastric cancer and indication of poor prognostic association with pathogens and microsatellite stability
Source: Genes Environ. 2021 Feb 10;43:3. doi: 10.1186/s41021-021-00174-6 (PMC7877109; doi:10.1186/s41021-021-00174-6)
Supplement: Supplementary file 3 — Additional file 3. [file 41021_2021_174_MOESM3_ESM.docx]

*Questionnaire for Epidemiological Study of Gastric Cancer*

Referring Dr:_______________ Hospital Name/No __________________________/______________

Referring Unit:______________ MZU Reg. No. /Date:_MZU/DBT/_____________

**PERSONAL HISTORY**

Hming (*Name)*: Mipa/Hmeichhia (*Male/Female*):

Kum (*Age):*  Tawng hman (*Language*): Nupui/pasal nei/neilo (*Marital status):* Pian ni*(Date of birth):*

*Nupui/pasal neiha kum zat* (*Age at the time of marriage*):

*Rihzawng (Weight):*  San zawng (*Height*):

Lehkha zir chen(*Education*): Eizawnna (*Occupation)*:

Unau engzat nge in nih*? (No. of Siblings):* [ ] Mipa (*Male*) [ ] Hmeichhia (*Female*) [ ]

Fa I nei em? (*Do you have children?):* Aw/*Yes* [ ] Aih/*No* [ ]

I neih chuan, fa engzat nge I neih? (*If yes, how many children do you have?):* [ ]

Mipa/Hmeichhia engzat nge? (*Gender of the children):*  Mipa(*Male)* [ ] Hmeichhia(*Female)* [ ]

(Thi sa a piang chhiar tel tur, chhiat erawh chhiar tel loh tur) (*Please include stillbirths; it is not necessary to include miscarriages)*

PermanentAddres:________________________________________________________________________________________________________________________________________________________________________________________________________________________________________________ ________________________________________________________________________PinCode______Tel/Mob.No.___________________________________________________________________________Email: _______________________________________

PresentAddres:______________________________________________________________________________________________________________________________________________________________________________________________________________________________________________________________________________________________________________________________________ PinCode__________Tel/Mob.No.__________________________________________________________ Email: ___________________________________________

Cancer Diagnosis/Treatment____________________________________________________________

Engtik kumah nge cancer I vei tih hmuhchhuah a nih? *(Year in which cancer was detected?):___________*

| Tumor | Site | Age | Histopathology | Surgery Date | Chemotherapy Date | Radiation |
| --- | --- | --- | --- | --- | --- | --- |
| 1^st^ Primary |  |  |  |  |  |  |
| 2^nd^ Primary |  |  |  |  |  |  |
| 3^rd^ Primary |  |  |  |  |  |  |

Syndrome Diagnosis:

|  |
| --- |

Consent for sample collection: Yes/No Date: _________________________

Blood collected: Yes/No Date: _____________ Received on_____________ From___________

Second sample collected: Yes/No Date:_____________Received by______________Thru____________

Tumor Tissue Collected: Yes/No Date_______________ Biorepository: Genesis Lab/MZU/ MSCI

Samples transmitted to MZU (sample type/ Date/ Method of transfer etc.)

Samples transmitted to NIBMG (sample type/ Date/ Method of transfer etc.)

Details taken by: _________________________________________ Date:_________________________

Pre- Test Counseling done by: _______________________________Date:_________________________

Post-Test Counseling done by: ______________________________Date:_________________________

**FAMILY INFORMATION:**

In chhungkua ah natna dang vei in awm em(cancer ni lo) ***(Any other type of diseases in the family (other than cancer)*:**

| Life style Habits |  |  |  |  |  |  |
| --- | --- | --- | --- | --- | --- | --- |
| Occupation |  |  |  |  |  |  |
| Disease Information |  |  |  |  |  |  |
| Sex/Age |  |  |  |  |  |  |
| Education |  |  |  |  |  |  |
| Relation |  |  |  |  |  |  |
| Name |  |  |  |  |  |  |

In chhungkua ah Cancer vei dang an awm em ***(Does anyone else in your family have cancer)***:

| Life style Habits |  |  |  |  |  |  |
| --- | --- | --- | --- | --- | --- | --- |
| Occupation |  |  |  |  |  |  |
| Disease Information |  |  |  |  |  |  |
| Sex/Age |  |  |  |  |  |  |
| Education |  |  |  |  |  |  |
| Relation |  |  |  |  |  |  |
| Name |  |  |  |  |  |  |

Hereditary: Yes [ ] No [ ] Autosomal Dominant: Yes [ ] No [ ]

Autosomal Recessive: Yes [ ] No [ ] Sex linked: Yes [ ] No [ ]

[ ] Cannot ascertain/Not applicable

[ ] Sporadic [ ] Early Onset [ ] Routine RET [ ] Familial

[ ] Others_________________

Chhungkaw member zat **(*Number of deaths in the family due to disease*)**:

Boral tawh (*Decease number*): [ ]

Boral chhan (Reason) – Pumpui cancer (*Gastric cancer*): [ ]; Adang (*Other* ): [

**PEDIGREE**

(Draw pedigree one degree above and below affected individuals and note consanguinity.)

**GEOETHNIC ORIGIN**

| Sub tribe |  |  |  |  |  |  |  |  |
| --- | --- | --- | --- | --- | --- | --- | --- | --- |
| Tribe |  |  |  |  |  |  |  |  |
| Occupation |  |  |  |  |  |  |  |  |
| Family name/Surname |  |  |  |  |  |  |  |  |
| Dist./State of origin |  |  |  |  |  |  |  |  |
| Present  place of stay (Dist./State) & duration |  |  |  |  |  |  |  |  |
| Place of birth  (Dist./State) |  |  |  |  |  |  |  |  |
|  | Index | Father | Mother | Paternal  Grandfather | Paternal  Grandmother | Maternal Grandfather | Maternal Grandmother | Remarks |

**Environmental/ Lifestyle Factors**

What has been your main occupation?__________________________________

| Hengah te hian hna I thawk em? I hnathawhnaah hetiang te hi I in chiahpiah tir em?  *(Do you have Occupational exposure to?)* | | No. of years | Age  (From / to) | Nature of use | Name of company/brand |
| --- | --- | --- | --- | --- | --- |
| Radiation (e.g. In a factory, laboratory/ medical setting) | Yes No  Don’t Know |  |  |  |  |
| Plastic factory/ burning/ | Yes No  Don’t Know |  |  |  |  |
| Tobacco plants / Rubber plant | Yes No  Don’t Know |  |  |  |  |
| Pesticides/  Pest control /  Mosquito Repellants | Yes No  Don’t Know |  |  |  |  |
| Chemical/Dyes/Fertilizer | Yes No  Don’t Know |  |  |  |  |
| Any other exposure  (Asbestos, Chromium or Lead) | Yes No  Don’t Know |  |  |  |  |
| Tuibur/ Local Alcohol preparation | Yes No  Don’t Know |  |  |  |  |

1. Was your mother an agriculture worker around the time of your birth? Yes/No
2. Has DDT ever been used in or around your household? Yes/No
3. What is your water supply source? River [ ] Tube well [ ] Govt./municipal [ ]
4. Other________________________________________________________________

I hna a hahthlak viau em, zan lam ah hna I thawk em(night duty)? (***Is your job stressful or do you perform shift work (night duty****)?)*: Aw/*Yes* [ ] Aih/*No* [ ]

In in bulah cell phone tower a awm em?*(Is there a cell phone tower near your house?)*:

Aw/Yes[ ] Aih/No [ ]

Exercise I la ngai em? *How often do you exercise?* Ngai lo(N*ever)* [ ]; Karkhatah vawi khat aia tlem(*Less than once a week)* [ ]; Karkhatah vawi khat(*Once a week)* [ ]; Karkhatah vawi 2-3 (*2-3 times a week)* [ ]; Karkhatah vawi 4-6 (*4-6 times a week)* [ ]; Nitin(*Everyday)* [ ]

**TASTE PREFERENCES**:

| **Do you consume**  **( I ei ngai em)** | **0 (Never)** | **1 (Little)**  **1 days in a week** | **2 (Average)**  **2-4 days in a week** | **3 (Heavy)**  **5-7 days in a week** |
| --- | --- | --- | --- | --- |
| Spicy food |  |  |  |  |
| Western food (Pizza,burgers,fries) |  |  |  |  |
| Burmies product |  |  |  |  |
| Sour test (tamarind, lime juice etc) |  |  |  |  |
| Bawngsa (*Beef)* |  |  |  |  |
| Vawksa (*Pork)* |  |  |  |  |
| Kelsa (*Mutton)* |  |  |  |  |
| Arsa (*Chicken)* |  |  |  |  |
| Artui (*Egg)* |  |  |  |  |
| Sangha (*Fish)* |  |  |  |  |
| fermented fish |  |  |  |  |
| Bekang/fermented pulse |  |  |  |  |
| Sa-Um |  |  |  |  |
| Extra salt with food |  |  |  |  |
| Pickles/chutneys |  |  |  |  |
| Smoked vegetables |  |  |  |  |
| Smoked meat |  |  |  |  |
| Fat intake |  |  |  |  |
| Boiled food |  |  |  |  |
| Fried food |  |  |  |  |
| Smoked food |  |  |  |  |
| Salt brand/type  (packed/raw) |  |  |  |  |
| Oil brand/type |  |  |  |  |
| Fibers food/fruits  (Banana/Bamboo shoots) |  |  |  |  |

*What type of utensils you normally use for your food items?)*: *Plastic* [ ] Aluminum [ ] Steel [ ] Other

Do you re-use oil for cooking/ frying: Aw/*Yes* [ ] Aih/*No* [ ]

Do you use Cosmetics/ Make up items: Regularly [ ] Occasionally [ ]

**Tobacco & alcohol History:**

| **Do you consume**  **( I ei ngai em)** | **0 (Never)** | **1 (Little)**  **1 days in a week** | **2 (Average)**  **2-4 days in a week** | **3 (Heavy)**  **5-7 days in a week** | **Av. Quantity per day** |
| --- | --- | --- | --- | --- | --- |
| Hnamdang siam (*Branded Alcohol)* |  |  |  |  |  |
| Mizo siam *(Local Alcohol)* |  |  |  |  |  |
| *Tuibur[*Bazar a lei (*Local)/* Mahni a siam (*Self-made)]* |  |  |  |  |  |
| Others |  |  |  |  |  |

Engtik atangin nge I in tan? (*When did you start taking alcohol?)* :

I nghei tawh anih chuan, engtik atangin? *(If quit already, since when?)*:

Engtik atangin nge I hmuam tan? (*When did you start taking tuibur?)*:

I nghei tawh anih chuan, engtik atangin? (*If quit already, since when?):*

If quantity of consumption of alcohol/ tuibur has changed during life time, the period of your highest consumption:

| Beverage (Name) | Yes/No | From age | To age | Av. Quantity per day | Days/ Week |
| --- | --- | --- | --- | --- | --- |
|  |  |  |  |  |  |
|  |  |  |  |  |  |
|  |  |  |  |  |  |

Mei I zu em? (*Do you smoke?):* Aw/*Yes* [ ] Aih/*No* [ ]

Engtik atangin nge I zuk tan? (*When did you start smoking?)*:

I nghei tawh anih chuan, engtik atangin? *(If quit already, since when?)*:

Has there ever been a time when you smoked at least one cigarette per day for three months or longer?

[ ] Yes [ ] No [ ] Don’t know

If yes, list consumption (excluding times when the subject did not smoke)

| Product | Yes/No | Used From/To | Frequency | Av. Quantity per day |
| --- | --- | --- | --- | --- |
| Cigarette(Brand name) |  |  |  |  |
| Biri |  |  |  |  |
| Zozial |  |  |  |  |

Vaihlo a siam thil dang tih I nei em? (*Do you consume other tobacco products?):*Aw/Yes [ ] Aih/No [ ]

Have you ever chewed pan or tobacco regularly? (At least once a week for six months or more) Yes [ ] No [ ] Don’t Know [ ]

| **Type** | **Yes/ No** | **From age** | **To age** | **No. per day** |
| --- | --- | --- | --- | --- |
| Chewing with tobacco and lime (khaini)  Pan+tabacco+betelnut+lime+catechu(mewa) |  |  |  |  |
| Gutka |  |  |  |  |
| Sahdah(*Oral snuff*) |  |  |  |  |
| Kuhva *(Pan/Beetle nut)* |  |  |  |  |
| Zarda Pan |  |  |  |  |
| Supari |  |  |  |  |
| Chewing without tobacco  (eg. pan without tobacco) |  |  |  |  |
| Adangte *(Others)* |  |  |  |  |

History of passive smoking:

Do any of your family member/colleagues smoke tobacco at home? Yes/No

Frequency of exposure to passive smoking: Rarely/ Continuously

**Medical History:**

I blood group eng nge? *(What is your* [*Blood group*](http://www.cancerresearchuk.org/health-professional/cancer-statistics/statistics-by-cancer-type/stomach-cancer/risk-factors)*?)*

A+ [ ] A- [ ] B+ [ ] B- [ ] AB+ [ ] AB- [ ] O+ [ ] O- [ ]

Ultrasonography:

Other: Region___________________________Report Date:_______________Impression___________

__________________________________________________________________________________________________________________________________________________________________________

CT scan: Region_____________________________________________Report Date________________

impression_________________________________________________________________________________________________________________________________________________________________

Colonoscopy/Endoscopy: Regions___________________________________Date__________________

Impression_________________________________________________________________________________________________________________________________________________________________

Surgery: Site/Procedure_______________________________________________________

Pathological Staging-pTNM___________________________________Date________________

Histopathological Report: Specimen_______________________Path No._________________

Date________________Impression_____________________________________________________________________________________________________________________________________________________________________________________________________________

IHC: Hormone receptor status

Tumor details: Specimen__________________________________Path No.________________

Report Date________________Grade_______________________Size of the tumor_____cm. Tumor emboli______________________________Lymphovascular Invasion____________

_____________________________________________________________________________

_____________________________________________________________________________

Other:________________________________________________________________________________________________________________________________________________

Treatment/Other Remarks:_______________________________________________________

**Syndromic features noted:**

Indigestion (Pum Puar) Nausea or vomiting (Luakchhuak) Dysphagia (Chawhelh)

Postprandial fullness (Hnawh ulh) Loss of appetite(Chaw ei tuilo) Melena(Ek dum)

Hematemesis (Thi a luak) Weight loss (Thla 6 chhunga kg8-10 vela tla hniam)

Pallor (Dawldang) Anaemia (Thisen nei tlem) Pain in abdomen(Pum na)

Natna/Damlohna dang I nei em? (*Do you have any other diseases?):* Aw/*Yes* [ ] Aih/*No* [ ]

I neih chuan, eng natna nge? (*If yes, what type of disease?):* _________________________________

*H. pylori* [ ] Diabetes [ ] obesity [ ] HIV [ ] HbsAg[ ] HCV[ ] EBV [ ] Gastric atrophy [ ] Others______________________

A hnuai ami te hmang hian enkawl I ni tawh em? History of taking HRT/[Reflux](http://www.cancerresearchuk.org/health-professional/cancer-statistics/statistics-by-cancer-type/stomach-cancer/risk-factors) /[Proton Pump Inhibitors/](http://www.cancerresearchuk.org/health-professional/cancer-statistics/statistics-by-cancer-type/stomach-cancer/risk-factors) Others(Give details)______

___________________________________________________________________________________________________________________________________________________________

**Obestric History :(Nau nei tawh zat)** Others

Gravity/Parity (Nau paizat)

Recurrent spontaneous abortions (Nau chhiat zat)

Still births/ Neonatal deaths (Thi a Hrin zat/Sen laia thi)

Congenital malformations (Fuke kim lova piang zat).

**Remtihna *(Consent):***

Heng a chunga thu te hi ka hriatpui a, ka biological sample hi zir chian atan pek ka remti thlap e.

*The information provided above was given with my full consent and I do not have any objection in providing my biological sample for research purposes. I have read and understood the consent information.*

Hmun*(Plac*e): Signature:

Date: Hming (*Name)*:

KA LAWM E

*(THANK YOU VERY MUCH FOR YOUR HELP)*

---------------------------------------------------------------------------------------------------------------------

Follow- Up Notes
